# Correction: Gender effects on outcomes of psychosomatic rehabilitation are reduced

**DOI:** 10.1371/journal.pone.0324362

**Published:** 2025-05-12

**Authors:** Juliane Burghardt, Manuel Sprung

**Notice of Republication**

This article [1] was republished on April 25, 2025, to update the author list in line with institutional recommendation, after an investigation by the Karl Landsteiner University of Health Science concluded that Friedrich Riffer did not meet the criteria for authorship. Please download this article again to view the correct version. The originally published, uncorrected article and the republished, corrected articles are provided here for reference.

## Supporting information

S1 File Originally published, uncorrected article. (PDF)

S2 File Republished, corrected article. (PDF)
