# Supplementary material for: Gender effects on outcomes of psychosomatic rehabilitation are reduced
Source: PLoS One. 2021 Aug 27;16(8):e0256916. doi: 10.1371/journal.pone.0256916 (PMC8396777; doi:10.1371/journal.pone.0256916)
Supplement: Attachment — Submitted filename: PLOS_2021.docx [file pone.0256916.s002.docx]

This is an interesting paper about the effects of gender on the treatment outcomes of inpatient psychiatric rehabilitation in Austria. The paper is fairly clear in the approach and analysis. The manuscript would benefit from a few changes for readability and clarity.

Introduction

*Please describe psychosomatic rehabilitation- how is this the same or different from psychiatric rehabilitation? It may be helpful to use either one term or the other- the term “psychiatric” is preferred to describe hospital treatment.

* Please use rehabilitation interventions not rehabilitations

p.4 Consider mentioning the subjective nature diagnosis – are there studies that examine the effect of clinician gender on treatment outcomes?

Method

*There is some duplication in description of study design.

*Please address why the BriefSCL and SCLwere both utilized and the comparability of results between them.

*It would be helpful for the reader to explain the “Hogrefe” system

Discussion

*How are differences in symptom distress related to diagnosis?Is there an interaction effect of diagnosis and QoL?

*It may be helpful to also consider measurement error for current or previous research findings. Is there gender bias in the standardized tools?

*The statement “The present findings confirm that gender-sensitive treatment is possible, even if women are still more distressed than men upon admission.” Is not well supported by your research question or results. Rather, it seems to suggest that rehabilitation interventions are effective despite limited access to gender-sensitive treatment. It may also suggest looking at measurement tools differently to capture gender differences and/or the use of qualitative exploration to reveal gender differences.

* There is also evidence that people in the age cohorts of younger and older adulthood may require age-specific treatment or gender-sensitive treatment based upon age.
